# Estimation and prediction of COVID-19 cases in Brazilian metropolises^*^

**DOI:** 10.1590/1518-8345.4501.3345

**Published:** 2020-06-26

**Authors:** George Jó Bezerra Sousa, Thiago Santos Garces, Virna Ribeiro Feitosa Cestari, Thereza Maria Magalhães Moreira, Raquel Sampaio Florêncio, Maria Lúcia Duarte Pereira

**Affiliations:** 1 Universidade Estadual do Ceará, Fortaleza, CE, Brazil.; 2 Scholarship holder at the Fundação Cearense de Apoio ao Desenvolvimento Científico e Tecnológico (FUNCAP), Brazil.; 3 Scholarship holder at the Coordenação de Aperfeiçoamento de Pessoal de Nível Superior (CAPES), Brazil.; 4 Scholarship holder at the Conselho Nacional de Desenvolvimento Científico e Tecnológico (CNPq), Brazil.

**Keywords:** Coronavirus Infections, Social Isolation, Forecasting, Epidemiology, Epidemiologic Models, Nursing, Infecções por Coronavirus, Isolamento Social, Previsões, Epidemiologia, Modelos Epidemiológicos, Enfermagem, Infecciones por Coronavirus, Aislamiento Social, Predicción, Epidemiología, Modelos Epidemiológicos, Enfermería

## Abstract

**Objective:**

to estimate the transmission rate, the epidemiological peak, and the number of deaths by the new coronavirus.

**Method:**

a mathematical and epidemiological model of susceptible, infected, and recovered cases was applied to the nine Brazilian capitals with the highest number of cases of the infection. The number of cases for the 80 days following the first case was estimated by solving the differential equations. The results were logarithmized and compared with the actual values to observe the model fit. In all scenarios, it was considered that no preventive measures had been taken.

**Results:**

the nine metropolises studied showed an upward curve of confirmed cases of COVID-19. The prediction data point to the peak of the infection between late April and early May. Fortaleza and Manaus had the highest transmission rates (≥2·0 and ≥1·8, respectively). Rio de Janeiro may have the largest number of infected people (692,957) and Florianópolis the smallest (24,750).

**Conclusion:**

the estimates of the transmission rate, epidemiological peak, and number of deaths from coronavirus in Brazilian metropolises presented expressive and important numbers the Brazilian Ministry of Health needs to consider. The results confirm the rapid spread of the virus and its high mortality in the country.

## Abstract (pt)

**Objetivo:**

estimar a taxa de transmissão, o pico epidemiológico e óbitos pelo novo coronavírus.

**Método:**

um modelo matemático e epidemiológico para os casos suscetíveis, infectados e recuperados foi aplicado nas nove capitais brasileiras com o maior número de casos da infecção. O número de casos para os 80 dias seguintes ao primeiro caso foi estimado por meio de equações diferenciais e os resultados foram colocados em logaritmos e comparados com os números reais para verificar o ajuste do modelo. Em todos os cenários, considerou-se que nenhuma medida preventiva foi tomada.

**Resultados:**

as nove metrópoles estudadas apresentaram uma curva de tendência de novos casos do coronavírus com um padrão ascendente. Os dados de predição apontam o pico da doença entre o final de abril e começo de maio. Fortaleza e Manaus apresentaram as maiores taxas de transmissão (≥2,0 e ≥1,8, respectivamente). Rio de Janeiro pode apresentar o maior número de pessoas infectadas (692.957) e Florianópolis a menor (24.750).

**Conclusão:**

as estimativas da taxa de transmissão, pico epidemiológico e óbitos pelo coronavírus nas metrópoles brasileiras apresentaram números importantes e expressivos a serem observados pelo Ministério da Saúde brasileiro. Os resultados confirmam a rápida disseminação do vírus e sua alta mortalidade no país.

## Abstract (es)

**Objetivo:**

estimar la tasa de transmisión, el pico epidemiológico y el número de muertes por el nuevo coronavirus.

**Método:**

se aplicó un modelo matemático y epidemiológico de casos susceptibles, infectados y recuperados a las nueve capitales brasileñas con mayor número de casos de infección. El número de casos para los 80 días siguientes al primer caso se estimó resolviendo las ecuaciones diferenciales. Los resultados fueron logaritmizados y comparados con los valores reales para observar el ajuste del modelo. En todos los escenarios, se consideró que no se habían adoptado medidas preventivas.

**Resultados:**

las nueve metrópolis estudiadas mostraron una curva ascendente de casos confirmados de COVID-19. Los datos de predicción apuntan al pico de la infección entre finales de abril y principios de mayo. Fortaleza y Manaus tuvieron las tasas de transmisión más altas (≥2·0 y ≥1·8, respectivamente). Río de Janeiro puede tener el mayor número de personas infectadas (692,957) y Florianópolis el menor (24,750).

**Conclusión:**

las estimaciones de la tasa de transmisión, el pico epidemiológico y el número de muertes por coronavirus en metrópolis brasileñas presentaron cifras expresivas e importantes que el Ministerio de Salud de Brasil necesita considerar. Los resultados confirman la rápida propagación del virus y su alta mortalidad en el país.

## Introduction

The new coronavirus (SARS-CoV-2) belongs to a family of viruses that cause diseases in the human respiratory system. Previous outbreaks of coronavirus (CoVs) include Severe Acute Respiratory Syndrome (SARS)-CoV and Middle East respiratory syndrome (MERS)-CoV as major threats to public health^(1)^.

The COVID-19 disease pandemic began in December 2019 in Wuhan, Hubei province, People’s Republic of China. It quickly spread to other Chinese provinces^(2)^. Due to its high spread rate, China declared COVID-19 a second-class infectious disease, with management measures for a first-class infectious disease (the most dangerous category of infection)^(3)^.

The spread of COVID-19 was rapid and global. The first confirmed case outside China was in Thailand, on January 13^th^, 2020. Next, cases of the disease were confirmed in Japan (January 16^th^); South Korea (January 20^th^); Taiwan and the United States (January 21^st^); Hong Kong and Macau from China (January 22^nd^); Singapore (January 25^th^); France, Nepal, and Vietnam (January 24^th^); Malaysia and Australia (January 25^th^); Canada (January 26^th^); Cambodia (January 27^th^); Germany (January 28^th^); Finland, United Arab Emirates and Sri Lanka (January 29^th^); Italy, India and the Philippines (January 30^th^); United Kingdom (January 31^st^)^(4)^, and the geographical expansion of this pandemic continues.

In this scenario, it remains to be established that the ongoing pandemic of COVID-19 is devastating, despite the extensive implementation of control measures. On January 30^th^, 2020, the World Health Organization (WHO) characterized the disease as a pandemic, being declared a Public Health Emergency of International Importance. As of April 1^st^, 2020, 823,626 cases of Covid-19 were confirmed, with 40,598 deaths worldwide^(5)^.

In an analysis of the COVID-19 case panel in Brazil until April 1^st^, 6,836 cases were confirmed by the country’s Ministry of Health, in addition to 241 deaths and a fatality rate of 3.5%. The cases are distributed throughout the national territory, with a greater concentration in the Southeast (4,223 cases; 62%), followed by the Northeast (1,007 cases; 15%), South (765 cases; 11%), Midwest (504 cases; 7%) and North (337 cases; 5%)^(6)^.

Brazilian data are alarming. In this sense, research is urgent to estimate the risk of this pandemic in Brazilian macro-regions. To know the most exposed urban centers that face the heaviest disease burden, it is imperative to closely monitor changes in epidemiology, the effect of public health strategies, and their social acceptance. Given the above, this research aimed to estimate the transmission rate, epidemiological peak, and the number of deaths by COVID-19 in the nine Brazilian capitals with the highest number of cases.

## Method

The first case of COVID-19 was diagnosed on February 27^th^, 2020 in São Paulo. On February 3^rd^, a public health emergency was decreed in the country, and on March 20^th^, 2020, community transmission of the disease was announced in the country^(7)^.

Thus, to understand this disease’s dynamics in the population, the SIR epidemiologic model proposed by Kermack and McKendric^(8)^ was applied. This model rests on the idea that there are three groups of individuals: susceptible (S), infected (I), and recovered (R). The mathematical expression of the model uses three differential equations, where β is the parameter that controls the transition between S and I, and γ is the parameter of the transition between I and R.

In this article, the first third of Brazilian capital cities with the highest number of cases until March 27^th^, 2020 was investigated, which corresponds to nine capital cities (out of twenty-seven). According to the Brazilian Institute of Geography and Statistics (IBGE), the estimated Brazilian population amounts to 208,494,900 inhabitants living in 5,571 cities, distributed in five great regions. The selected capitals were Belo Horizonte, São Paulo and Rio de Janeiro in the Southeast region; Curitiba, Florianópolis and Porto Alegre in the South; Manaus in the North; Salvador and Fortaleza in the Northeast. Brasilia, in the Midwest, would be added, but was removed from the pool of investigated cities due to difficulties to find official data.

Data were extracted from the daily epidemiological reports since the first day of confirmed cases until March 30^th^, 2020 and the capital’s population was according to the IBGE^(9)^. Initially, graphs were created with the actual number of confirmed cases in each city until the end date, followed by their logarithmic transformation to show growth patterns. Then, the differential equations were solved for each of the nine scenarios and the number of cases was predicted until the 80^th^ day of infection since day one. To test the model’s fit, the natural logarithm of the observed and predicted number of cases was used. They were graphically presented for the sake of a better understanding.

Moreover, the disease’s basic reproduction rate (R_0_) could be identified, which shows how many healthy people an ill person can infect. The predicted number of cases, the day of peak, and the possible number of deaths were also investigated, considering the maximum number of people who can be sick and 1% lethality. These values were considered as if no prevention measure had been taken. Data were analyzed in R software, using the package deSolve.

This work did not require Ethics Committee approval because the state health department freely distributed the data on the internet. Yet, the authors complied with Brazilian resolution 466/2012 on ethics for research.

## Results

In total, 2,829 confirmed cases of COVID-19 were analyzed in nine Brazilian capital cities. In the Southeast, São Paulo (SP), Rio de Janeiro (RJ), and Belo Horizonte (BH) notified the first cases on February 22^nd^, March 6^th^, and March 16^th^, respectively. Until the end of data collection, there were notified 1,233 cases in SP, 553 in RJ, and 163 in BH.

**Figure 1:**
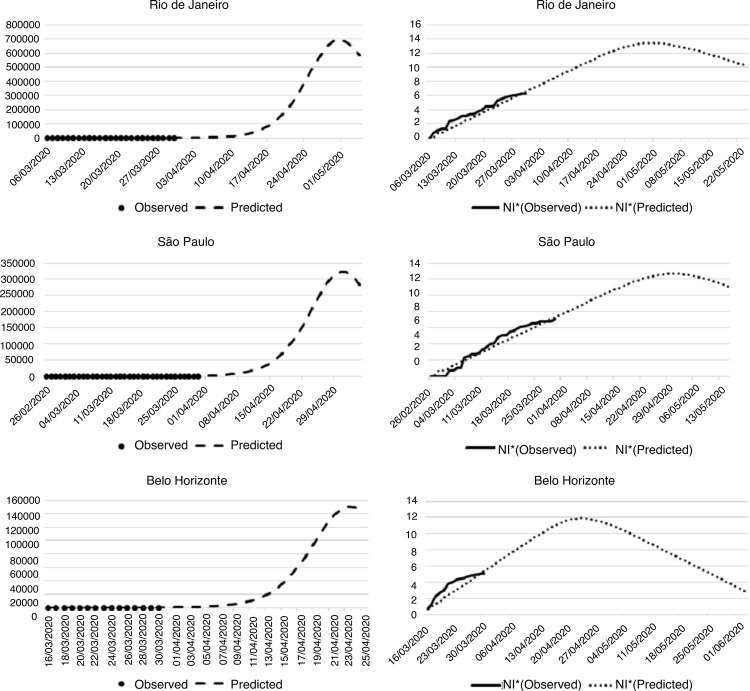
Projection of the number of people infected by the new coronavirus in the cities of the Southeast Region. Fortaleza, CE, 2020 ^*^Nl = Natural logarithm

Regarding the number of predicted cases, the observed cases were superior to the modeled number. In BH, some degree of stability was observed since day 14, with the number of cases remaining inferior to the model (Figure 1).

In the South Region, Curitiba, Florianópolis and Porto Alegre notified the first cases as from March 5^th^ (Porto Alegre) and March 12^th^ (Curitiba and Florianópolis). Until March 30^th^, the number of confirmed cases increased to 77 in Curitiba, 50 in Florianópolis, and 143 in Porto Alegre.

**Figure 2:**
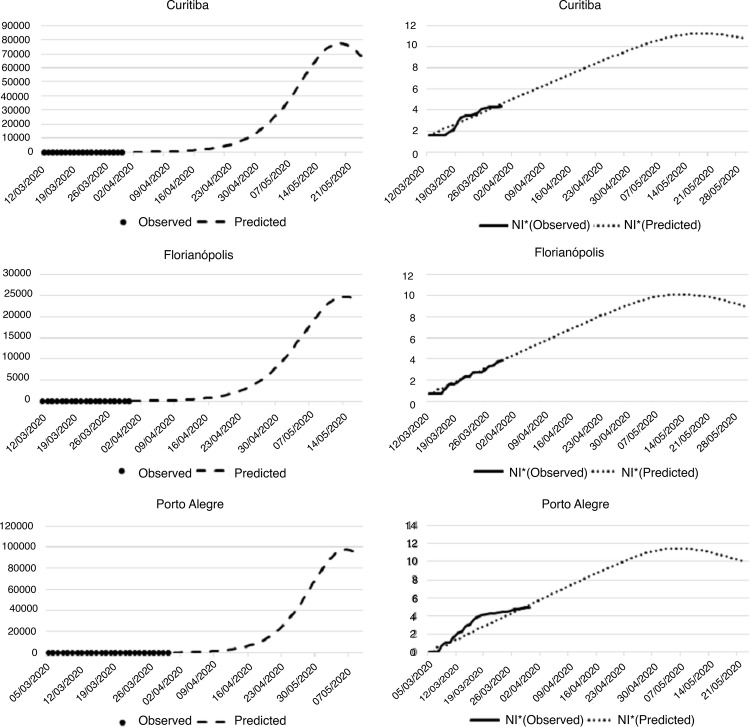
– Projection of the number of infected people by the new coronavirus in the cities of the South Region. Fortaleza, CE, Brazil, 2020 ^*^Nl = Natural logarithm

The cases estimated with the help of the SIR model in Curitiba, Florianópolis, and Porto Alegre showed that the observed and predicted case numbers were similar (Figure 2). Porto Alegre presented more observed cases than the model until the twentieth day of infection. From that point, the number of observed cases remained relatively constant and inferior to the model.

**Figure 3:**
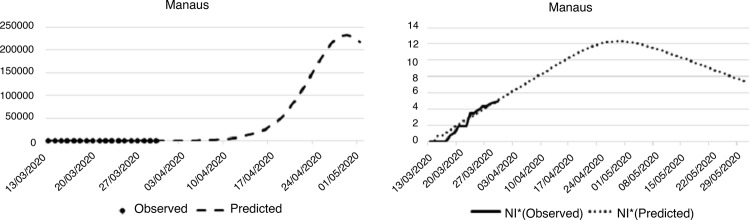
– Projection of the number of infected people by the new coronavirus in the capital of the North Region. Fortaleza, CE, Brazil, 2020 ^*^Nl = Natural logarithm

In the North Region, Manaus was the only city investigated. The first case of COVID-19 in the city was registered on March 13^th^ and, on the last day of the investigation, 140 cases had been registered. The sharp increase in the number of cases was similar to the model, as shown in Figure 3.

**Figure 4:**
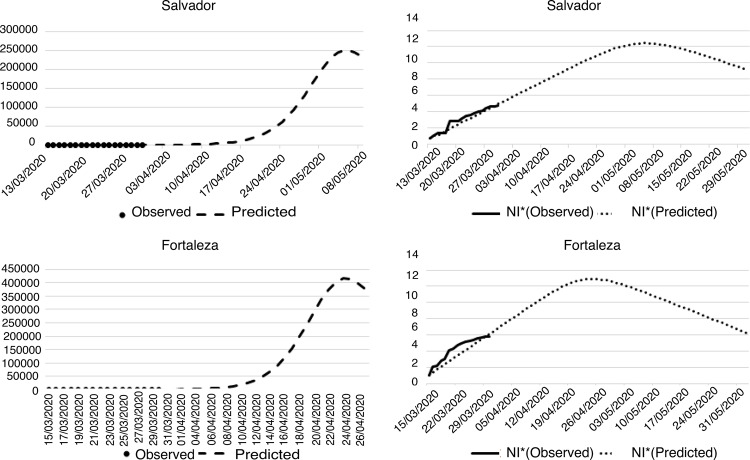
– Projection of the number of infected people by the new coronavirus in the cities of the Northeast Region. Fortaleza, CE, Brazil, 2020 ^*^Nl = Natural logarithm

In the Northeast Region, Salvador and Fortaleza notified the first cases on March 13^th^ and 16^th^, respectively. The number of confirmed cases until March 30^th^ was 117 in Salvador and 382 in Fortaleza. In the predictive model, Salvador and Fortaleza presented a trend similar to the model (Figure 4).

Finally, Table 1 presents the numerical results of the outcomes predicted for COVID-19 in the capital cities under study. The prediction data appoint the occurrence of the infection peak between the end of April and the beginning of May. Fortaleza and Manaus have the highest transmission rates (≥2·0 and ≥1·8, respectively). Rio de Janeiro may have the largest number of infected people (692,957) and Florianópolis the smallest (24,750).

**Table 1:** – Prediction of the new coronavirus outcomes. Fortaleza, CE, Brazil, 2020

Capital	Transmission Rate	Number of days from the first case to the peak	Peak date	Maximum number of infected people	Maximum number of deaths
Belo Horizonte	1·50	39	April 23	151,087	1,510
Curitiba	1·40	69	May 19	77,303	773
Florianópolis	1·42	64	May 14	24,750	247
Fortaleza	2·01	41	April 23	414,946	4,149
Manaus	1·86	48	April 29	231,401	2,314
Porto Alegre	1·53	63	May 6	97,830	978
Rio de Janeiro	1·75	56	April 30	692,957	6,929
Salvador	1·67	55	May 06	249,435	2,494
São Paulo	1·29	65	April 30	323,018	3,230

## Discussion

Brazil is marked by intense socioeconomic inequalities and health conditions, with relevant geographical differences, whether in the levels of health risks or the access to the resources available in the country’s health system^(10)^. Endemics, epidemics, and pandemics have historical and social roots, and their main determinants are the poor living conditions of the population, the different forms of spatial occupation, and the lack of access to services.

Unlike other infectious diseases, COVID-19 affects developed and developing countries, making no social distinction. The results of this investigation showed the highest number of cases of COVID-19 in the Southeast region, distributed in the cities of SP and RJ, considered the largest and most developed national metropolises and, to a lesser extent, in BH. São Paulo is the main financial, corporate, and mercantile center in South America, being the most influential Brazilian city at the global level. RJ is the largest international tourist destination in Brazil, a characteristic that led to the appearance of the first case of the disease in the country.

Also in the Southeast, BH showed an upward pattern of the disease curve. In comparison with SP and RJ, however, it presented a lower number of cases, which can be justified by having a smaller population than the two other cities, and by the more uniform distribution of basic health services and their scope. In BH, human resources, health actions, and services offered by the Unified Health System are distributed to offer quality services, with greater equity and easy and timely access to meet the needs of a larger portion of the population^(11)^, which can contribute to the early diagnosis and containment of disease cases.

Moving on, the South region, with the cities of Curitiba, Florianópolis and Porto Alegre, had a lower number of cases. These cities represent a major tourist, economic, and cultural center with European influence, evidenced in its predominantly elderly population. People living in cities in the South region have greater access to health services when compared to other Brazilian regions^(12)^.

It is worth noting that the unequal distribution of COVID-19 cases among Brazilian regions is also influenced by underreporting. The North and Northeast regions are marked by a worse assessment of the health status, greater restriction of activities, and lesser use of health services, despite the greater coverage of public programs^(13)^.

Representing the North region, Manaus, the state capital of Amazonas, is the main financial, corporate and commercial center of the region. It is the most populous city, with 2.1 million inhabitants and one of the largest tourist destinations in Brazil. The growth of cases in the city is expressive and linked to a social scene where asymmetry, verticality, competitiveness, and weak relations between the cities prevail, in addition to an insufficient health service network with difficulties to maintain human resources^(14)^.

In the Northeast region, the cases of COVID-19 were analyzed in Salvador and Fortaleza, cities with a great international travel flow. Salvador is the most populous city in the Northeast and the third in the country. Fortaleza, the state capital of Ceará, has the highest demographic density among the Brazilian capitals. In these cities, an exponential growth pattern of cases was observed and Fortaleza also presented the highest transmission rate. Currently, Fortaleza concentrates 91% of COVID-19 cases in the state of Ceará, concentrated in neighborhoods of different social and economic levels.

In this context, the number of national and international tourists who seek Brazil as a destination has increased every year. Capitals such as São Paulo, Rio de Janeiro, Curitiba, Salvador, and Fortaleza figure among the most popular destinations for tourists, whether for their natural landscapes, culture, or economic attractions. To accommodate this number of tourists, Brazil has become one of the emerging countries with the greatest potential for the development of air transport.

This is due to the country’s territorial dimension and high geographical and social mobility of its population, the accelerated displacement of economic frontiers, Brazil’s competitive insertion in global markets and monetary stability, and the increased purchasing power for consumers^(15)^. The establishment of hubs in several airports has enhanced the entrance of foreigners in different countries of the world.

The economic advantages of tourism in Brazil are undeniable, however, the issues of travel and health are an existing concern. The profiles of travelers differ in terms of origin and destination, which can directly influence the occurrence of epidemics and pandemics, often of unexpected magnitude and severity^(16)^, such as COVID-19. Also, limited coverage and access to health services in the country can corroborate the spread of diseases.

While a small portion of the Brazilian population has access to health services, many people face a decrease in the availability of hospital beds^(17)^. This factor, linked to COVID-19’s pandemic potential, put the response capacity of epidemiological surveillance services at the center of attention and required preventive measures from the Brazilian government, such as confinement and social distancing.

Extensive measures are needed to reduce the interpersonal transmission of COVID-19^(1)^. Some of the measures adopted, such as spraying disinfectant and alcohol in the air, on roads, vehicles, and people have not been effective though^(3)^. More expanded measures include isolation of cases, identification and monitoring of contacts, environmental disinfection, and use of personal protective equipment^(4)^. Regarding the control strategies, social distancing stood out as a strategy that limits human-to-human transmission, as well as reducing secondary infections between close contacts and health professionals, preventing transmission amplification events, and reducing or delaying the dissemination of the virus.

Moreover, epidemics and pandemics paralyze the economic, social, political, and cultural development, interfering in the demographic trajectory of the locations where they spread^(18)^. The emergence of COVID-19 and its consequences has left the worldwide population with feelings of fears, concerns, and anxiety, which can further expand the disease data^(19)^.

The biological, mental, emotional, social, and economic chaos caused by COVID-19 requires a quick response from the federal government and open and effective communication with state governors. In Brazil, divergences are observed between members of the state and federal governments as to the best measures to adopt to face this crisis. This lack of understanding is factual and may result in a greater number of deaths, as a result of the characteristics of the virus and unequal access to the health system and health technologies in the country.

The publication of the expected number of cases in the investigated metropolises in Brazilian journals is new. This evidence allows managers to organize health services, based on public policies such as the creation of hospital beds, purchasing of medical equipment, and development of health education actions to assure quarantine or social distancing.

The main limitation of this study arises from the use of a secondary database, as data for some cases were incomplete. Furthermore, underreporting and/or insufficient testing might influence the predicted peak. It is also important to highlight that the results did not consider social distancing measures that aim to reduce the transmission rate of the virus.

## Conclusion

The estimates of the transmission rate, epidemiological peak, and number of deaths by COVID-19 in the Brazilian metropolises presented expressive and important numbers which the Brazilian Ministry of Health should take into account. All metropolises showed an exponential growth in the number of cases. The transmission rate was higher in Fortaleza and in Manaus, where many deaths are expected. Thus, the results confirm the rapid spread of the virus and its high mortality in Brazil.
